# Evidence for a Role for the Dorsal Anterior Cingulate Cortex in Disengaging from an Incorrect Action

**DOI:** 10.1371/journal.pone.0101126

**Published:** 2014-06-26

**Authors:** Eldad Yitzhak Hochman, Avinash Rao Vaidya, Lesley K. Fellows

**Affiliations:** Department of Neurology and Neurosurgery, Montreal Neurological Institute, McGill University, Montreal, Quebec, Canada; University of California, Davis, United States of America

## Abstract

Adjusting for an error requires both disengaging from the wrong course of action and initiating a corrective response. The dorsal anterior cingulate cortex (dACC) has been implicated in both these processes in the decision-making and action monitoring literatures. Here, we aimed to distinguish between these putative functions with a variant of the Eriksen flanker task that manipulated response requirements (i.e. one or two finger responses). We found that two event-related potentials originating from the dACC (error-related negativity (ERN) and anterior N2) only reflected the representation of the incorrect response: these waveforms were larger when the incorrect response involved two fingers rather than one finger. The increase in ERN magnitude was also accompanied by a reduction in spontaneous error corrections. These results argue that activity in the dACC reflects a process involved in disengaging from an ongoing incorrect action, clearing the way for the correct response.

## Introduction

To err is human, and to quickly correct those errors can be critical for survival. The dorsal anterior cingulate cortex (dACC) is involved in guiding action when the dominant, stimulus-driven response is not appropriate. This is demonstrated in patients with damage to this region, who have difficulty overcoming pre-potent responses and initiating movements that are not stimulus-driven [1], [2]. More recent lesion work points to a specific role of dACC in rapid corrective responses [3]–[5], and in adapting response speed based on past experience [6]. Thus, this region appears to play a key role in adjusting behavior after a mistake [7]. However, the mechanism through which the dACC acts is not clear. A different literature has shown that dACC activity measured with fMRI or electrophysiology increases during periods where animals and humans must deviate from a ‘default’ response to initiate novel actions [8], [9]. Studies using foraging paradigms suggest that the dACC becomes more active as disengaging from the default option becomes more desirable [10]–[13].

Correcting an incorrect action and disengaging from a decision option with diminishing returns might rely on a common process. In both cases, there is a need to disengage from a pre-potent response (whether induced within trial by a misleading stimulus, or encouraged by a recent history of rewards), clearing the way for a new action. However, it is not clear how the dACC enables such shifts. dACC might control disengagement from the incorrect action [14], play a role in the initiation of the new action [15]–[17], or perhaps both.

Two frontocentral event-related potentials (ERPs) associated with action selection are commonly thought to reflect dACC function: a negativity preceding correct responses (anterior N2), and the error related negativity (ERN/Ne) following incorrect responses [18]–[21]. The amplitude of the ERN has been shown to co-vary with the speed and likelihood of error correction [22], [23], and the subsequent slowing of correct responses following an error [21], [22] (although see Hajcak, McDonald and Simons [24]). While the significance of this activity is hotly debated, most current theories agree that these waveforms are functionally linked to error correction and subsequent adaptations [14], [25], [26].

Recently, we found that the ERN was sensitive to the representation of the incorrect response in a flanker paradigm [14]. This potential was larger when motor errors were committed with a dominant hand, or with fingers with more refined control. Interestingly, these errors were also followed by fewer and slower corrections. This finding suggested that the dACC contributes to disengaging from an undesirable pattern of responding and that the amplitude of the ERN reflects the intensity of that process. However, that study could not address whether the dACC operated on the incorrect response alone, or also acted to facilitate the subsequent correction, as these two factors were not independently controlled.

**Figure 1:**
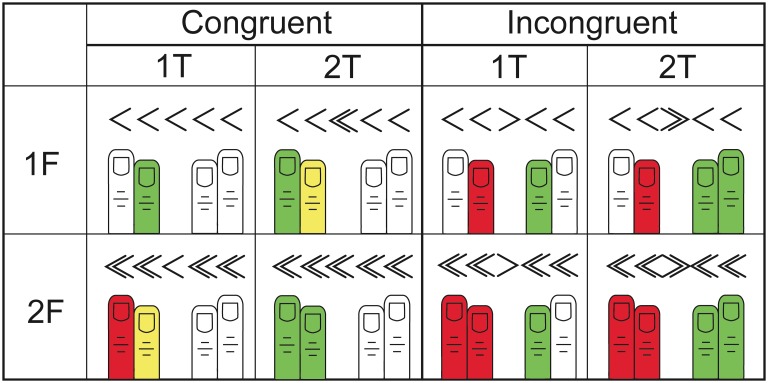
Experimental design. The stimuli (target and flankers) in each type of trial in the incongruent and congruent direction conditions are shown along with cartoons of the corresponding fingers involved. Target (correct) responses (green) with one (1T) or two (2T) fingers were indicated by a single arrowhead or double arrowhead target stimulus, respectively. Similarly, incorrect (flanker) responses (red) with one (1F) or two (2F) fingers were induced by single arrowhead or double arrowhead flanker stimuli, respectively. Yellow indicates fingers that were involved in both the error and correct responses, and thus did not have independent representation. White indicates fingers that were not involved in either response option.

In the current study, we independently manipulated the motor representations of response alternatives through the number of fingers involved in a given response. We asked participants to complete a four-choice arrowhead flanker task, requiring responses with their right or left index and middle fingers together, or with their right or left index fingers alone (see Fig. 1). Movements involving only the index finger recruit the motor cortex representations of this digit, whereas simultaneous movements of both index and middle fingers recruit the motor representations of both [27]–[30]. Flanker and target stimuli could indicate one or two-finger responses on either hand. Participants encountered all possible pairs of response alternatives. Critically, this parametric manipulation allowed us to test whether ERP activity originating from the dACC was sensitive to the representation (one or two fingers) of the incorrect response induced by the flanker, the representation of the correct response indicated by the target, or some interaction between the representations of both responses.

We hypothesized that error-related dACC activity reflects disengagement from the incorrect (flanker-induced) response, clearing the way for a correct response, analogous to its role in flexibly disengaging from a pre-potent response in multi-trial foraging contexts. This hypothesis predicts greater dACC activity and fewer error corrections when the motor representation of the incorrect response is more extensive (i.e. involves two fingers together, compared to one finger alone). In contrast, if the dACC is involved in the initiation of the correct response, we would expect the ERN and anterior N2 to be sensitive to the representation of the correct (target) response, so these waveforms would be larger when the correct response involved two fingers. A final alternative is that the dACC might react to both response representations, suggesting a dual role in disengaging from the incorrect response and producing the correct response.

## Materials and Methods

### Ethics statement

The McGill University Research Ethics Board approved the study protocol and all participants gave written informed consent.

### Participants

Twenty-three participants volunteered for this study. Two met exclusion criteria (self-report of any neurological or psychiatric disorders, major head trauma or recent regular use of psychoactive drugs), and three were lost to attrition. Of the 18 remaining participants (10 females, mean age 22.31 years, SD = 5.44), 13 were right-handed, three left-handed and two had no hand preference, as evaluated using the Edinburgh Handedness Inventory [31].

### Materials

The experiment was programmed using E-Prime 1.1 (Psychology Software Tools, Inc., Pittsburgh, PA, USA). Stimuli were presented on an 18-inch monitor (Dell Inc., Round Rock, TX, USA) and responses were recorded using a standard North American English keyboard (Orbyx Electronics, Canada).

### Procedure

Participants performed a speeded, four-choice, arrowhead version of the Eriksen flanker task [32] in which they were asked to respond with either their right or left index fingers alone (one-finger response), or their right or left index and middle fingers together (two-finger response), depending on the target stimulus. Subjects used the ‘X’ and Z’ keys on the left hand and the ‘N’ and ‘M’ keys on the right hand for their index and middle fingers, respectively. Two-finger responses were only considered as a single response if the inter-finger latency of these responses was less than 30 ms. This threshold is within the latency of the fastest corrections [33], ensuring that such two-finger actions could be considered a single response. Only erroneous responses that matched the hand and number of fingers represented by the flanker stimulus were considered for the main analysis of the behavioral and electrophysiological data, as we could not be sure of the source of other, non-flanker induced errors. The frequency of errors that did not match the flanker in each condition is reported in Table 1. Correct and corrective responses were similarly only of interest if they matched the hand and number of fingers represented by the target stimulus.

**Table 1:** Behavioral Data.

	Congruent				Incongruent				Statistical Comparisons
	1F/1T	1F/2T	2F/1T	2F/2T	1F/1T	1F/2T	2F/1T	2F/2T	
**Correct Response** **Frequency** **(%)**	87.73(4.0)	62.64(8.3)	68.58(6.4)	81.48(10.0)	62.31(9.0)	56.02(7.6)	69.32(7.8)	51.54(8.1)	1F/1T(C)>1F/1T(I)^a^ 2F/2T(C)>2F/2T(I)^a^ 1F/1T(I)>2F/1T(I)^a^ 2F/1T(I)>1F/2T(I)*1F/1T(I)>1F/2T(I)*2F/1T(I)>2F/2T(I)*
**Missed Response** **Frequency** **(%)**	4.31(3.0)	5.69(3.7)	7.45(3.58)	3.56(2.3)	10.31(5.0)	12.84(6.0)	12.15(6.8)	14.27(6.7)	1F/1T(I)>1F/1T(C)^#^2F/2T(I)>2F/2T(C)^a^ 2F/1T(I)>1F/1T(I)*2F/2T(I)>1F/2T(I)*1F/2T(I)>1F/1T(I)*2F/2T(I)>2F/1T(I)∧
**Non-Flanker** **Error** **Response** **Frequency** **(%)**	7.73(3.0)	3.13(2.0)	2.54(1.9)	14.83(8.3)	2.48(1.6)	21.03(3.9)	13.55(4.2)	20.18(6.9)	
**Correct Response** **RT (ms)**	296.07(20.8)	311.65(20.8)	315.49(23.5)	288.56(19.5)	339.57(26.4)	345.91(24.0)	344.95(26.4)	347.24(25.3)	1F/1T(I)>1F/1T(C)^a^ 2F/2T(I)>2F/2T(C)^a^ 2F/1T(I)>1F/1T(I)*1F/2T(I)>1F/1T(I)*
**Erroneous Response** **RT** **(ms)**	-	281.21(20.2)	262.06(16.2)	-	281.72(18.7)	266.56(21.4)	238.99(18.6)	263.79(20.3)	1F/1T(I)>2F/1T(I)^a^ 1F/1T(I)>1F/2T(I)^#^2F/2T(I)>2F/1T(I)^ a^

Values represent means with SDs in parentheses. Congruency refers to whether the target and flankers were pointing in the same (congruent) or opposite (incongruent) direction. The terms 1F and 2F refer to the number of fingers represented in the flanker, while 1T and 2T refer to the number of fingers represented in the target. ‘C’ refers to the congruent direction condition, and ‘I’ refers to the incongruent direction condition. Symbols indicate *P*-value for the difference as assessed using Tukey’s HSD tests. ∧*P* = .06, *P<.05, #*P*<.005, ^a^ *P*<.0005. Post-hoc comparisons for the 1F/2T and 2F/1T conditions in the congruent direction condition were avoided due to potential confounding inter-finger conflict interactions.

Arrowheads pointing left indicated a left-hand response and arrowheads pointing right, a right hand response. A single arrowhead target required a one-finger response (1T), whereas two closely stacked arrowheads required a two-finger response (2T; Fig. 1). Flankers consisted of two evenly spaced arrowhead stimuli on either side of the target (one arrowhead (1F), or two arrowheads (2F)). Stimuli were balanced across conditions for congruency of the hand represented in the flanker and target responses, and the two levels of response representation (one or two-finger responses) for both the flanker and target. Target and flanker arrowheads were presented pointing left and right in an equal number of trials in each condition.

The experiment was split into two sessions, each subdivided into eight blocks. Each block consisted of 192 trials with 24 trials per condition. Blocks were spaced to give subjects time to rest before continuing the task; time between sessions ranged from 4 to 29 days. Before each session, subjects completed a practice session under experimenter supervision with a minimum of 128 practice trials to ensure that they understood the task.

In each trial, subjects were presented with a fixation cross in the center of the screen for 500 ms, which was followed by the flanker array for 80 ms. The target stimulus then appeared for 15 ms in the center of the screen, in the middle of the flanker array. The target and flanker stimuli were both generated using a 30 pt Courier New font. The screen was then cleared for the duration of the response window, which was dynamically adjusted based on the subjects’ accuracy (as in Fiehler, Ullsperger and von Cramon [34]) for a target error frequency of 30% on incongruent direction trials, using the following equation R*_n_*_+1_ = R*_n_*±(R*_n_* [.15|T_E_–T_IE/_T_I_|]). Where R_n_ is the duration of the response window on trial *n*, T_E_ is the target error frequency, T_IE_ is the total number of errors on incongruent direction trials and T_I_ is the total number of incongruent direction trials. The window had a baseline length of 450 ms at the beginning of each block and increased as subjects’ accuracy on incongruent direction trials dropped below the target rate of 70% to a maximum of 525 ms, and decreased to a minimum of 350 ms as accuracy improved above 70%. Late responses or failures to respond also increased the length of the response window independent of accuracy using the same equation with a T_E_ of 0%, and where T_IE_ was equal to the number of missed responses on incongruent direction trials.

Each trial was followed by a feedback stimulus for 600 ms: a green square indicating that the subject was responding fast enough, or a red square indicating that the subject failed to respond in time (a miss). At the end of each block, subjects were given a tally of their total errors and misses in the block to encourage vigilance throughout the session. Error correction was not encouraged throughout the task because we were interested in the effect of response representation on the corrective response and assumed that instructing participants to correct their errors would introduce confounding elements into corrective behavior.

### Electrophysiological recording and analysis

EEG was recorded with 128 electrodes using the Geodesic system (Electrical Geodesics Inc., Eugene, OH, USA). All channels were referenced to the Cz electrode during data acquisition, and impedance was kept below 50 kΩ, as recommended for the amplifier used. The filter band-pass was 0.01–100 Hz and the EEG was digitized at 256 Hz. EEG data were processed and analysed using BrainVision Analyzer software (Brain Products, GmbH, Munchen, Germany). EEG data were re-referenced to the averaged mastoids and band-pass filtered between 0.1–30 Hz, 12 dB/octave. Bad channels were identified by visual inspection and estimated using Hjorth’s nearest neighbours method (Hjorth 1975). EOG artefacts were corrected within BrainVision using the Gratton and Coles algorithm [35]. For the ERN and the correct response-related negativity (CRN, Gehring and Knight [36], Scheffers and Coles [37], Vidal, Hasbroucq, Grapperon and Bonnet [38]) epochs were time-locked −150 ms to 800 ms around the response and baseline corrected using the average activity from −150 to −50 ms. Epochs with artefacts (voltage steps exceeding ±50 µV; low activity criterion of 0.5 µV, difference criterion of 100 µV within a 100 ms segment) were automatically rejected. Epochs were averaged together and activity at FCz (E6) and six immediately adjacent electrodes (E5, E7, E12, E13, E107 and E113) were further averaged to produce a cleaner, more representative signal at a frontocentral region of interest (ROI). The ERN was defined at this ROI as the absolute minimum amplitude between −50 to 150 ms around erroneous and correct responses, respectively. The CRN was defined in the same ROI and time window as the minimum local peak. Local peak values were chosen for analysis of the CRN, as this negativity was much smaller than the ERN. Difference waves were calculated by subtracting the average voltage in correct responses from erroneous responses. The same peak detection method was used for defining the ERN in difference waves as was applied to the raw ERN waveform. We also analyzed both the raw ERN waveform and the difference wave using the mean voltage between −10 and 90 ms around the response at the same FCz centered ROI.

The raw and difference waveforms corresponding to the late error positivity (Pe, see Overbeek, Nieuwenhuis and Ridderinkhof [39] for a review) were defined as the mean amplitude from 250–500 ms after an erroneous response at an ROI consisting of Pz (E62) and six immediately adjacent electrodes (E54, E55, E61, E68, E79 and E88). As with the ERN, difference waves were computed by subtracting the voltage in waveforms following correct responses from erroneous responses.

The anterior N2 is commonly measured as the difference between the waveforms in the incongruent and congruent direction conditions to remove the effects of slow stimulus processing activity on these waveforms. However, our manipulation had the potential for interactions between direction congruency and the representation of the target and flanker responses (one vs. two fingers). Thus, we chose to remove the effects of slow, stimulus related potentials on the anterior N2 by band-pass filtering the EEG data between 3–30 Hz. In order to avoid effects of RT on the latency of the anterior N2, we locked the component to the response and analyzed it in the pre-response period, as in Nieuwenhuis, Yeung, van den Wildenberg and Ridderinkhof [40]. This approach reduces the temporal variance of this component due to differences in RT, and makes it easier to measure differences in this potential in a pre-defined time-window. Epochs were created from −300 to 300 ms around the correct response and were baseline corrected using the average activity from −100 to 0 ms before the appearance of the flanker. The anterior N2 was defined as the average voltage and the difference in the local minimum amplitude and the preceding local maximum amplitude (i.e. peak-to-peak value) between −150 to −50 ms at the same FCz centered ROI used for the ERN and CRN. Peak-to-peak values were used to avoid small differences in baseline values that could influence our results. The position of the Geodesic sensor net equivalents of FCz 10-10 system was based on Luu and Ferree [41].

### Source Analysis

Cortical EEG source imaging was performed on the averaged ERN and anterior N2 waveform components of individual subjects, in each condition, using Brainstorm [42]. Cortical currents were mapped to a distributed source model consisting of 15,002 elementary current dipoles. Dipole locations and orientations were fixed to the surface of the standard Montreal Neurological Institute brain (Colin27) and the EEG forward model of volume currents was completed with a symmetric boundary element model (BEM) generated with OpenMEEG [43], [44], also available through Brainstorm’s user interface. An inverse model of EEG sources was obtained using the standard weighted minimum-norm current estimate available in Brainstorm (wMNE; Baillet, Mosher and Leahy [45]). The magnitude of each individual source time series was rectified, and source activity at each cortical location was standardized using the z-score transformation with respect to the average and standard deviation of the source activity during baseline: a 100-ms time window in the pre-response period. For the ERN, the baseline was defined as −150 to −50 ms prior to the response, and −250 to −150 ms prior to the response for the anterior N2 component. Parcellation of the cingulate cortex into anatomical ROIs was derived in Brainstorm from the atlas by Destrieux, Fischl, Dale and Halgren [46]. Regions of the cingulate cortex were concatenated bilaterally to define the rostral anterior cingulate cortex (rACC), dACC and posterior cingulate cortex (PCC) bilateral ROIs. Within the nomenclature used by Destrieux, Fischl, Dale and Halgren [46], the rACC was defined as the subcallosal and anterior cingulate gyrus and sulcus, the dACC as the middle anterior and middle posterior cingulate gyri and sulci, and the PCC as the posterior dorsal and posterior ventral parts of the cingulate gyrus. Source activity within these ROIs was estimated by averaging the activity of elementary sources within each ROI: the average activity within each ROI was taken within the same time windows as those used for examining the mean voltage in the EEG sensor data.

### Statistical analysis

Correct response frequency, RT on correct responses, missed response (i.e. failure to respond within the response window) frequency and the amplitude of the anterior N2 were analyzed using three-way within subject analyses of variance (ANOVA). Direction congruency (congruent or incongruent target and flanker direction), the size of the flanker response (one or two fingers) and the size of the target response (one or two fingers) were treated as the three factors. Error frequency, RT on erroneous responses and the magnitude of the ERN, CRN and Pe were analyzed using two way within-subject ANOVA with target and flanker response size as the two factors in the incongruent direction condition only. We did not analyze errors in the congruent direction condition, as the target and flanker were identical in these conditions. Correct and erroneous responses in the congruent direction 1F/2T and 2F/1T trials also shared features within the same hand and therefore did not have independent representations in target and flanker, and were thus ignored in post-hoc tests to avoid difficulties in interpretation. Tukey’s HSD tests were used for all parametric post-hoc comparisons. Correction frequency was analyzed with a non-parametric two way Friedman’s test using the estimated χ^2^ statistic and the same factors used for error-related measures, with Wilcoxon signed rank post-hoc tests adjusted for multiple comparisons. Source activity for the ERN was compared using a one-way, within-subjects ANOVA between ROIs, collapsing across the size of the correct and erroneous response. The same analysis was used for comparing source activity of the anterior N2, but only including waveforms from the incongruent direction condition, given the small size of the anterior N2 ERP in the congruent direction condition.

## Results

### Behavioral data

The experiment had three main factors: target-flanker direction congruency (congruent–same direction, incongruent–different directions), the correct (target) response (1 or 2 fingers) and the incorrect (flanker) response (1 or 2 fingers) (Fig. 1). We do not report post-hoc comparisons for trials in the congruent direction conditions where the responses represented by the target and flanker differed in the number of fingers involved (i.e. 1F/2T, 2F/1T). Our hypothesis does not make predictions for these conditions, where target and flanker responses share features within a hand, and presumably have motor representations that are nested, since the one finger response is subsumed in the two finger response. However, data from these conditions are provided to give a complete profile of participants’ performance.

**Figure 2:**
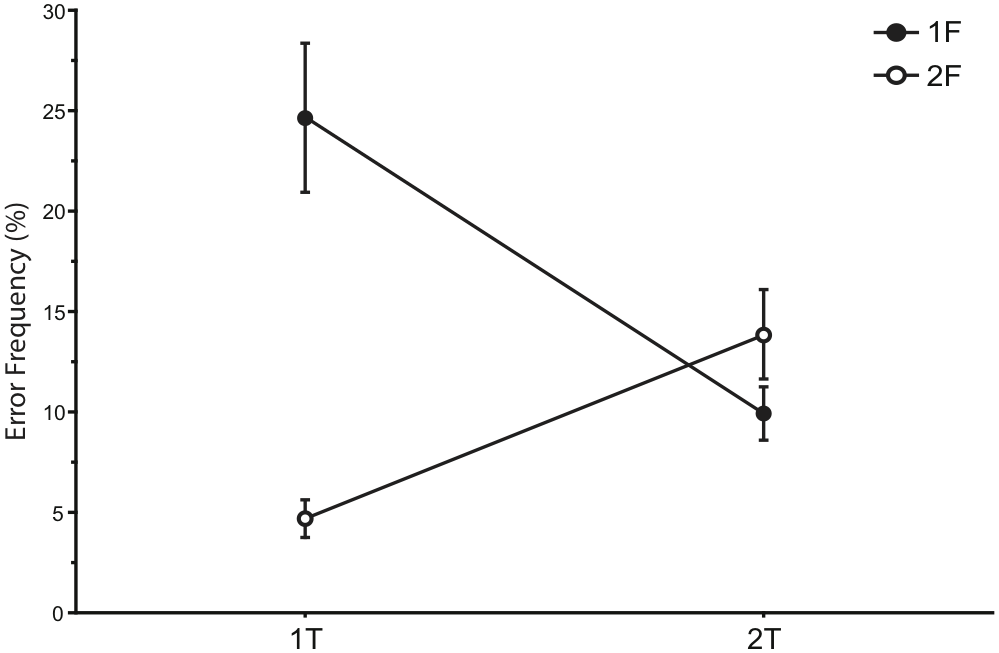
Error frequency in the incongruent direction condition at each level of response representation: one-finger response represented in the target (1T) and two-finger responses represented in the target (2T), as well as one-finger response represented in the flanker (1F) and two-finger response represented in the flanker (2F). Error frequency increased when the representation of the responses in the target and flanker were of the same size (1F/1T and 2F/2T) compared to when the representation of these responses was different (1F/2T and 2F/1T). The figure only includes errors where the erring response matched the response represented by the flanker stimulus. Error bars represent 1 SD.

We first examined error frequency in the incongruent direction condition to evaluate whether error rate was influenced by the manipulation of the number of fingers indicated by the target and flanker (Fig. 2). An interaction between target and flanker responses was found in the frequency of erroneous responses produced (*F*_1,17_ = 103.63, *P*<.00001), with more frequent errors when the number of fingers indicated by the target and flanker stimuli was the same (1F/1T and 2F/2T) than when they were different (1F/2T and 2F/1T; *P*’s<.02). Within the congruent direction condition, errors were committed in a mean 20.59% (SD = 6.8%) of 2F/1T trials, and a mean 28.89% (SD = 9.1%) of 1F/2T trials. As the same response was represented in the target and flanker on 1F/1T and 2F/2T trials in the congruent direction condition, errors in these trials were not induced by the flanker and thus did not meet the criteria for our analysis.

**Figure 3:**
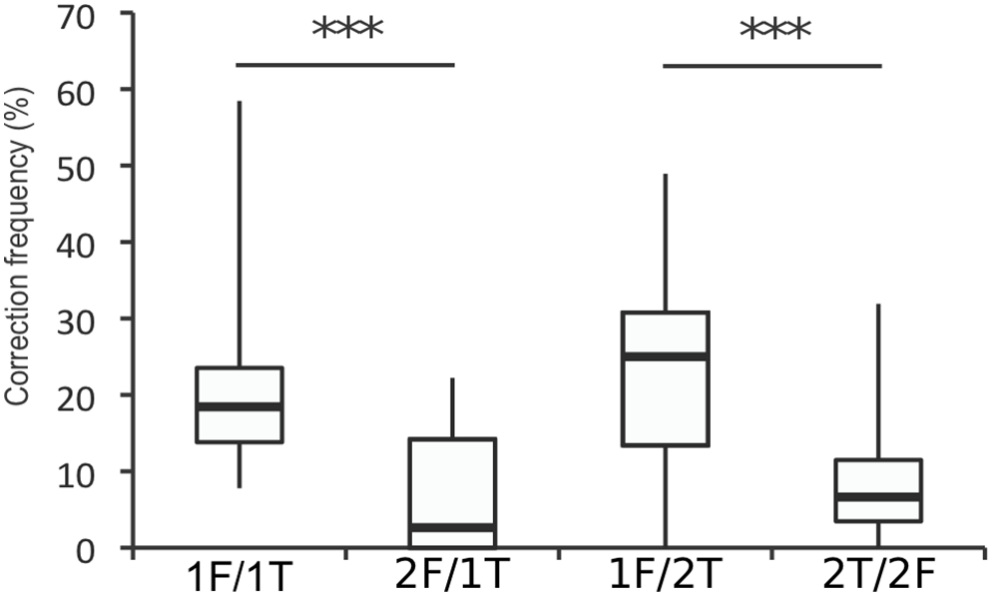
Box plot of the frequency of uninstructed corrective responses in the incongruent direction condition. Corrections were more frequent following errors in 1F trials, independent of the representation of the correct response. Whiskers represent the range, the solid black bar represents the median, ***P<.001, Wilcoxon signed rank post-hoc tests corrected for multiple comparisons.

Unlike error frequency, correction frequency was only affected by the flanker response, not the similarity of response alternatives (Fig. 3). As with error responses, we focused on corrections that followed errors where the incorrect response was the response indicated by the flanker. We only analyzed corrections following incongruent errors, as the corrective response required execution of the target response with the opposite hand, and was thus independent from the incorrect response. Within the incongruent condition, there was a significant main effect for the flanker response (χ^2^_3_ = 14.2, *P*<.01), with no interaction with, or main effect for, the target response, (χ^2^_3′_s≤0.22, *P*’s>.1). More corrections were produced following one-finger errors (1F/1T and 1F/2T) than following two-finger errors (2F/1T and 2F/2T) (1F/1T vs. 2F/1T: W_18_ = 9, *P*<.001; 1F/2T vs. 2F/2T: W_17_ = 5, *P*<.001). The low frequency of corrective responses in the 2F/1T and 2F/2T conditions meant that the reaction times of corrective responses could not be compared. Within the congruent direction condition, the median correction rate on 2F/1T trials was 0% (range: 0–2.08%), whereas the median correction rate in 1F/2T trials was 46.48% (range: 23.59–76.83%).

Data on reaction times and the frequency of correct and missed responses are summarized in Table 1. We found a three-way interaction between direction congruency and the responses represented by the flanker and target stimuli on correct response RT, (*F*_1,17_ = 80.30, *P*<.00001). Within the direction congruent condition, correct response RT was longer for trials when the target and flanker responses were different (1F/2T, 2F/1T) compared to trials where they were identical (1F/1T, 2F/2T). In trials where the direction of the target and flanker were incongruent, correct response RT was longer for 1F/1T and 2F/2T compared to the equivalent trials in the congruent direction condition. Within the incongruent direction condition, correct response RT did not consistently vary with the flanker or target response, nor with the similarity of responses. Thus, correct response RT was not consistently affected by the number of fingers indicated by the target or flanker, or by the similarity of the target and flanker responses.

We analyzed the frequency of correct responses to further evaluate how changing the number of fingers indicated by the flanker and target affected accuracy. A three way interaction was found between direction congruency and the flanker and target responses (*F*_1,17_ = 151.76 *P*<.00001). Correct responses were more frequent when the direction of the target and flanker were congruent than when they were incongruent when the size of the response alternatives was the same (i.e. 1F/1T and 2F/2T trials). Within the incongruent direction condition, 1T correct responses were more frequent on 2F than 1F trials, but there was no effect of flanker response on the frequency of 2T correct responses.

We also evaluated the frequency of missed responses (i.e. failure to respond by the deadline) as an additional indicator of task performance. There was a three-way interaction between direction congruency and the flanker and target responses (*F*_1,17_ = 40.14, *P*<.0001). Misses were less frequent in the congruent direction condition than in the incongruent direction condition. Within the incongruent direction condition, missed responses were more frequent when two fingers were represented in target or flanker stimulus, with no effect of the mismatch between response alternatives.

We assessed whether errors were more pre-potent in any particular condition by comparing the RT of erroneous responses, previously proposed as an index of pre-potency [47]. An interaction was found between the representation of the response alternatives (*F*_1,17_ = 72.45, *P*<.00001). However, post-hoc comparisons between different levels of target and flanker responses revealed no consistent effects.

In summary, manipulating the similarity of the number of fingers indicated by the target and flanker stimuli induced more errors, but had less clear effects on other measures of task performance. There was a clear effect of direction congruency on the frequency of correct and missed responses, as well as on the RT of correct responses.

### Event-related potentials

Due to the low frequency of errors in the 2F/1T condition, two participants with fewer than six artifact free error epochs were excluded from the analysis of the ERN. This minimum has previously been found to be sufficient for reliable measurement of the ERN for within-subject comparisons [48]. However, we also performed the same analysis using a minimum of 15 error epochs, excluding six subjects, and found a similar pattern of results to those described below.

**Figure 4:**
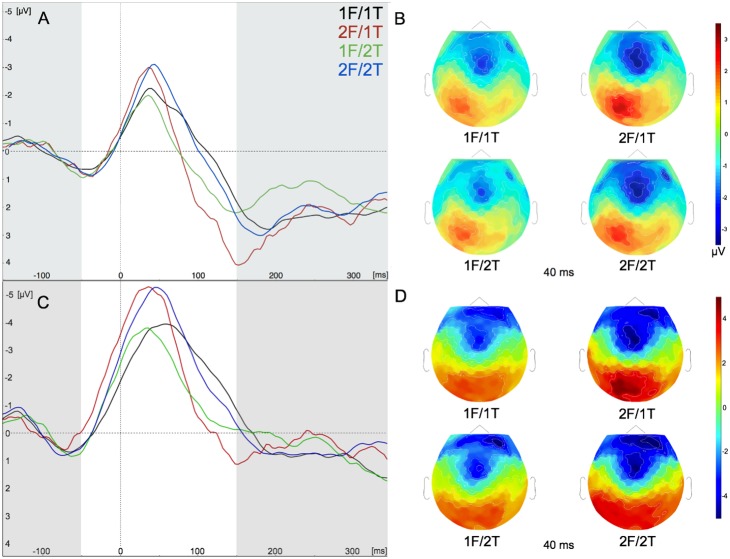
The amplitude of the error-related negativity (ERN) was increased when two fingers were represented in the flanker, whereas the representation of the response in the target had no effect on the amplitude of the ERN. Response alternatives were defined by the number of fingers represented in the target and flanker: one-finger flanker (1F) and one-finger target (1T), or two-finger flanker (2F) and two-finger target (2T). (**A**) Grand-average raw error waveforms from −150 to 350 ms locked to the response at the FCz ROI in the incongruent direction condition. (**B**) Scalp topographies of the grand-average raw waveforms in each condition at 40 ms after the response. (**C**) Grand-average error-correct difference waveforms locked to the response at the FCz ROI in the incongruent direction condition. (**D**) Scalp topographies of the grand-average difference waveforms in each condition at 40 ms after the response. Highlighted timeframe on grand-averaged waveforms represents the window used for detecting the peak amplitude of the ERN.

We were primarily interested in the effects of our manipulation of the number of fingers indicated by the target and flanker stimuli on the magnitude of the ERN and anterior N2. Namely, whether these waveforms would reflect the representation of either the correct (target) or incorrect (flanker) response alternative, or some combination thereof. Analysis of the peak amplitude of the raw ERN waveform (Fig. 4A) showed a main effect for the flanker response (*F*_1,15_ = 13.66, *P*<.01). The ERN was larger in 2F/1T and 2F/2T conditions than in the 1F/1T and 1F/2T conditions, respectively (*P*’s<.05). There was no significant main effect or interaction with the target response (*P’s>.*1). The same pattern was found for the peak amplitude of the difference wave computation of the ERN (Fig. 4C), with a main effect for the flanker response (*F*_1,15_ = 10.62, *P*<.01), and larger peak ERN amplitude in conditions with a larger flanker response representation (*P*’s<.05). Again, there was no significant interaction with, or main effect for, the target response (*P*’s>.1).

We also examined the mean voltage of the raw ERN waveform and the difference wave to determine the reliability of our findings using different methods of analysis. Analysis of the raw waveform showed a significant interaction between the target and flanker responses (*F*_1,15_ = 3.25, *P*<.05). However, post-hoc tests found that effects of the target response were not significant (*P*’s>.06). The ERN was larger in the 2F/2T condition compared to 1F/2T (*P*<.005), but no effect was seen for the flanker response at the 1T level (*P*>.1). An analysis of the mean voltage of the difference waveform showed a significant main effect for the flanker response (*F*_1,15_ = 14.36, *P*<.002), with no interaction or main effect for the target response (*F*’s_1,15_ ≤ 1.09, *P*’s>.1). Post-hoc tests showed that the mean voltage of the ERN was larger in the 2F condition compared to the 1F condition, regardless of the target response (*P*’s<.05). Thus, across several analytic approaches the ERN was mainly affected by the number of fingers indicated by the flanker stimulus, without any consistent effect of the number of fingers indicated by the target stimulus.

**Figure 5:**
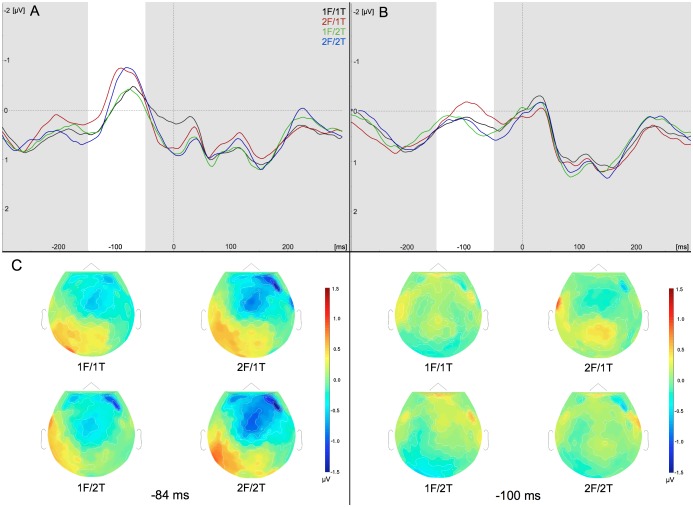
The amplitude of the anterior N2 was higher in the incongruent direction condition when two fingers were represented in the flanker. Response alternatives were defined by the number of fingers represented in the target and flanker: one-finger flanker (1F) and one-finger target (1T), or two-finger flanker (2F) and two-finger target (2T). (**A**) Grand-average waveforms from −300 to 300 ms locked to the response at the FCz ROI in the incongruent direction condition. (**B**) Grand-average waveforms from −300 to 300 ms locked to the response at the FCz ROI in the congruent condition. (**C**) Scalp topographies of the grand-average waveforms in the incongruent direction condition at 84 ms before the response. (**D**) Scalp topographies of the grand-average waveforms in the congruent condition at 100 ms before the response. Highlighted timeframe on grand-averaged waveforms represents the window used for detecting the peak amplitude of the anterior N2.

We similarly tested the effects of direction congruency and target and flanker response alternatives on the peak amplitude of the anterior N2 preceding correct responses (Fig. 5). The amplitude of this waveform was sensitive to the number of fingers indicated by the flanker, as well as direction congruency, but not to the number of fingers indicated by the target. Testing the peak amplitude of the anterior N2 revealed a significant interaction between the flanker response and direction congruency (F_1,17_ = 6.17, *P*<.05). However, the three-way interaction between direction congruency and flanker and target responses, and two-way interactions between the target and flanker responses, and between direction congruency and the target response were all non-significant (*F*’s_1,17_≤1.31, *P*’s>.1). Post-hoc comparisons between direction congruency levels showed that the anterior N2 was significantly larger in the incongruent direction condition of 2F/2T trials (*P*<.0005), though not in 1F/1T trials (*P*>.1).

Crucially, we examined the effect of the flanker response on the anterior N2 within the incongruent direction condition. Like the ERN, the anterior N2 was larger on 2F trials, independent of the target response within the incongruent direction condition (i.e. 1T/2F and 2T/2F trials) (*P*’s*<*.01). No significant effects were seen for the flanker response in the congruent direction condition (*P*’s>.1). Thus, the peak amplitude of the anterior N2 was affected by the flanker response, but not by the target response.

We also examined the mean voltage of the anterior N2 to determine if the same pattern of results would be seen with a different method of analysis. As with the peak voltage, the interaction between direction congruency and the number of fingers indicated by the flanker was significant (*F*’s_1,17_ = 4.87, *P*<.05). There was no significant three-way interaction between direction congruency and the target and flanker responses, nor between direction congruency and the target response, or between the target and flanker responses (*F*’s_1,17_≤4.23, *P*’s>.05). Post-hoc tests found that the mean voltage of the anterior N2 was larger in the incongruent direction condition than the congruent direction condition in both 1F/1T and 2F/2T conditions (*P*’s<.02). Within the incongruent direction condition, the anterior N2 was larger in the 2F condition at both 1T and 2T levels (*P*’s<.05). This pattern of results was very similar to that seen for the peak amplitude of the anterior N2, with clear effects for direction congruency and the flanker response, but not the correct response.

**Figure 6:**
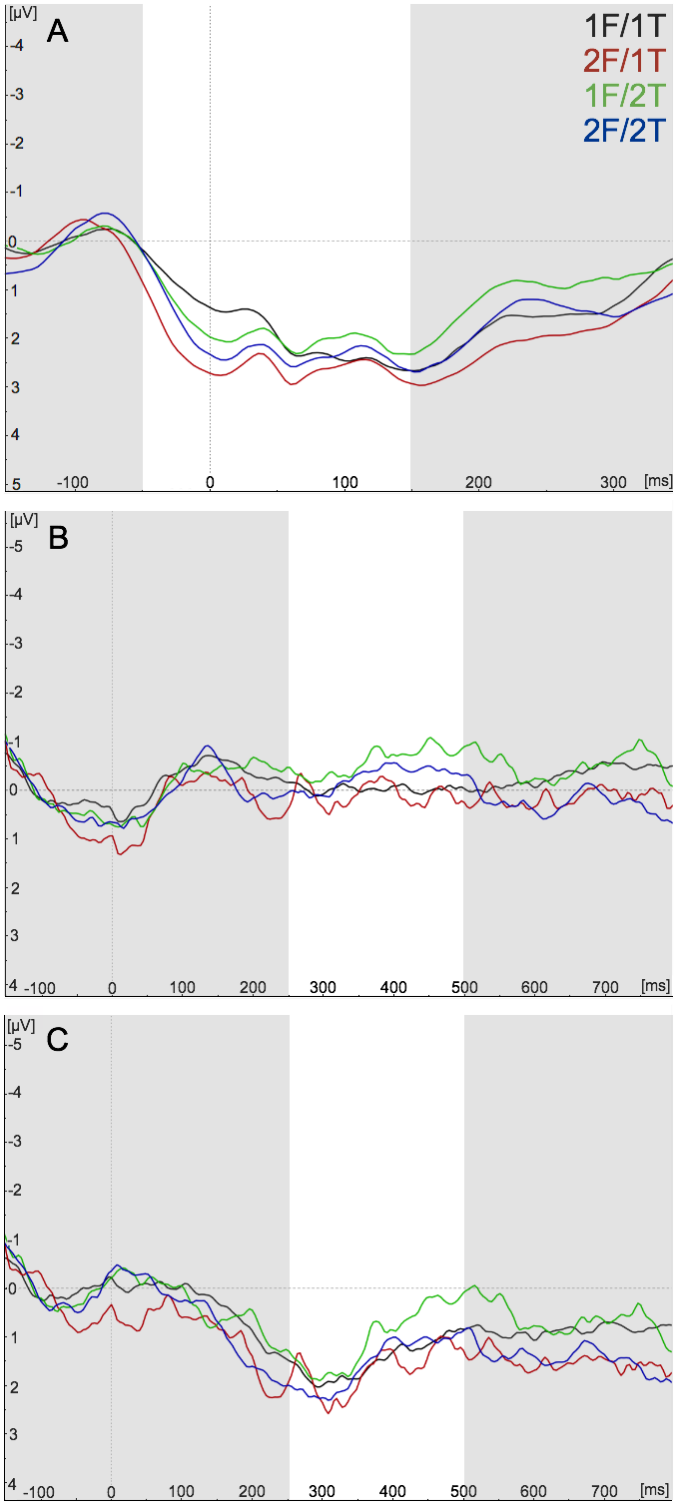
The correct response-related negativity (CRN) and error-related positivity (Pe) did not respond to the manipulation of the representation of response alternatives. Response alternatives were defined by the number of fingers represented in the target and flanker: one-finger flanker (1F) and one-finger target (1T), or two-finger flanker (2F) and two-finger target (2T). (**A**) Grand-average raw correct waveforms from −150 to 350 ms locked to the response in the incongruent condition at the FCz ROI. (**B**) The raw error waveforms from −150 to 800 ms locked to the response in the incongruent condition at the Pz ROI. (**C**) Grand-average error-correct difference waveforms from −150 to 800 ms locked to the response in the incongruent condition at the Pz ROI. Highlighted timeframe on grand-averaged waveforms represents the window used for detecting the peak amplitude of the CRN, and the mean voltage of the Pe.

To determine whether the effect of the number of fingers indicated by the flanker was specific to the ERN and anterior N2, or common to other response locked waveforms, we also examined how our manipulation affected the CRN and the Pe (Fig. 6). Analysis of the peak amplitude of the CRN did not reveal any significant interaction between the target and flanker response, or any main effect for the target response (Fig. 6A) (*F*’s_1,16_≤2.21, *P*’s>.1). There was a trend toward a significant effect for the flanker response (*F*_1,16_ = 3.94, *P* = .06). Post-hoc tests showed that the CRN was significantly larger in 1F/1T compared to 2F/1T trials (*P*<.05). However, there was no comparable difference in the CRN between 1F/2T and 2F/2T trials (*P*>.4). Hence, the pattern of the CRN results differed from the ERN findings, indicating that the observed effects of the flanker response were specific to the ERN following errors and the anterior N2 preceding correct responses.

Both the raw (Fig. 6B) and the difference waveform (Fig. 6C) of the late Pe were not sensitive to our manipulation of the representation of response alternatives, (*F*’s_1,15_≤1.99, *P*’s >.1). Overall, this waveform did not react to the number of fingers indicated by the target or flanker stimuli, or to their interaction.

### EEG source analysis

**Figure 7:**
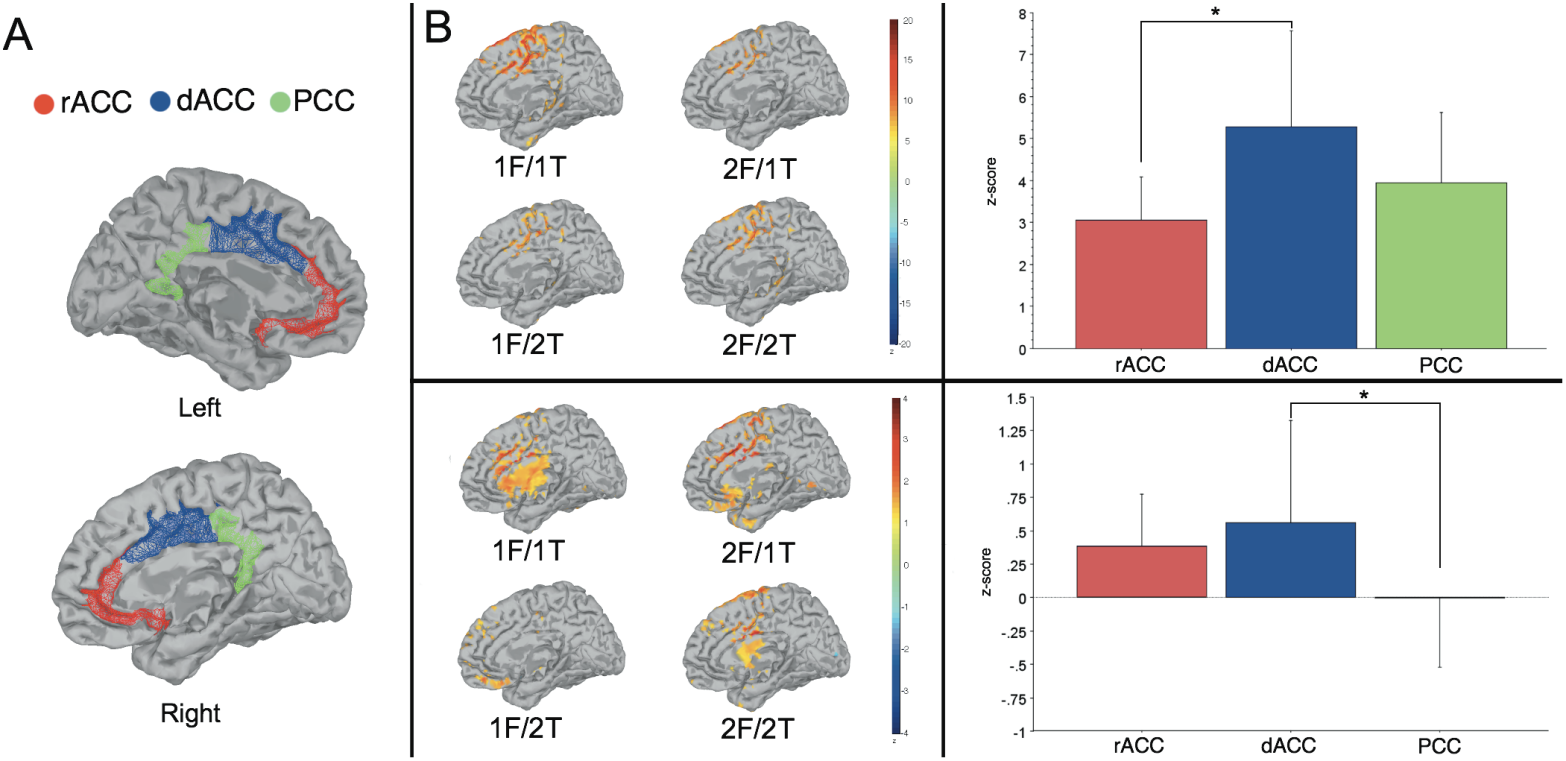
Source analysis of the error-related negativity (ERN) and anterior N2 revealed that source activity was highest in the dorsal anterior cingulate cortex (dACC) for both EEG components. (**A**) The regions of interest (ROIs) chosen for the parcellation of the cingulate cortex are shown in the sagittal plane in the right and left hemispheres. The rostral anterior cingulate cortex (rACC) is shown in red, the dACC in blue and the posterior cingulate cortex (PCC) in green. (**B**) Source activity of the ERN at 40 ms after the response represented at each dipole mapped to the cortical mantle in the sagittal plane. The color bar represents the *z*-score value of the source activity as measured from a baseline from −150 to −50 ms before the response. Source activity is shown for the incongruent direction condition for each pair of response alternatives: one-finger error (1F) and one-finger correct (1T), and two-finger error (2F) and two-finger correct (2T). (**C**) Source activity of the anterior N2 at 84 ms before the response in the incongruent direction condition. The color bar represents the *z*-score value of the source activity as measured from a baseline from −250 to −150 ms before the response. (**D**) Average z-scored source activity of the ERN at each ROI from −10 to 90 ms around the time of the response, collapsed across response alternative conditions. (**E**) Average z-scored source activity of the anterior N2 at each ROI from −150 to −50 ms around the time of the response, collapsed across response alternative conditions. *P<.05, Tukey’s HSD tests.

The distributed source model of cortical currents determined whether the anterior N2 and ERN ERPs shared the same brain generator (Fig. 7). We compared source activity in the rostral ACC (rACC), dACC and posterior cingulate cortex (PCC) (Fig. 7A) during the time window of the ERN and anterior N2. For the ERN, a significant effect was found for ROI, (*F*_2,15_ = 9.443, *P*<.001) with the highest activity in the dACC (Fig. 7D). Post-hoc tests showed that this activity was significantly higher in the dACC compared to the rACC (*P*<.01), though no significant difference was observed between the dACC and PCC, or between the rACC and PCC (*P*’s>.05). The dACC also appeared to be the focus of the anterior N2. The effect of ROI was near significance (*F*_2, 17_ = 2.991, *P* = .06), with the highest activity within the dACC (Fig. 7E). Post-hoc tests revealed that source activity was significantly higher in the dACC compared to the PCC (*P*<.05), though no significant differences were observed between the dACC and the rACC, or between the PCC and the rACC (*P*’s>.05). Thus, both waveforms appeared to share a common generator in the dACC.

## Discussion

Rapid adjustment after a mistake is central to the flexible control of actions. The dACC appears to have an important role in this process, being critical for the rapid initiation of corrective responses [3]–[5]. However, prior work has not established whether dACC is critical for disengaging from the incorrect response, producing the corrective response, or both. Here, we addressed this issue with a design that independently manipulated the representation of two response alternatives, asking whether dACC-related activity and error correction were sensitive to the motor representation of responses indicated by the target and flanker. Both the ERN on error trials, and the anterior N2 on correct trials, reacted only to the representation of the incorrect response. There was no significant effect of the correct response representation, and no interaction between the representations of the correct and incorrect responses. Further, corrections were less frequent when the representation of the incorrect response was larger, while there was no effect of the representation of the correct response on this measure.

Several recent studies have suggested that dACC activity reflects the tendency to disengage from one action in favor of another in multi-trial decision-making contexts [10]–[12], and dACC lesions disrupt value-based action selection, again in multi-trial contexts [49], [50]. Our findings suggest that dACC activity similarly reflects disengagement from an incorrect action, here on a millisecond timescale, within-trial in a choice RT task. Electrophysiological studies in monkeys indicate that disengagement-related dACC activity in foraging contexts is time-locked within-trial to action execution [10], much like the ERPs examined here, consistent with this view.

In the context of foraging, dACC activity becomes more intense as abandoning the default option becomes more desirable [10], [12], [13]. Analogously, the disengagement process might need to be more intense when the response to be disengaged has a larger representation. In line with this notion, Shenhav, Botvinick and Cohen [26] have suggested that dACC might set the intensity of the control process to disengage from the incorrect response. Disengagement from an incorrect action might be necessary for clearing the way for subsequent alternative responses. Here, we found that corrections were less frequent when the motor representation of the incorrect response was larger (two fingers, as opposed to one finger), a condition also associated with an increased ERN, similar to the findings of Hochman, Orr and Gehring [14]. These results support the notion that the disengagement process reflected in this component affects the ability to quickly shift to an alternative action. The finding that dACC damage impairs rapid corrections in similar choice RT tasks also supports this interpretation [3]–[5]. We did not find evidence that the dACC directly influences the correct response, at least in this choice RT context, for example by expediting or ‘energizing,’ actions [15], [16], or by controlling response initiation [17].

The anterior N2 might reflect a process similar to the ERN, but preceding correct responses. Correct responses are often preceded by partial errors, revealed by electromyographic activity in an erring muscle that does not reach the threshold for a full response [51]. The anterior N2 is linked to the occurrence of these partial errors, much like the ERN is linked to the occurrence of a full error [52], [53]. The current findings support the notion that the anterior N2 reflects a similar process as the ERN, and likely originates from a common generator in the dACC [20]. We suggest that this process enables disengagement from an incorrect response to allow a correct response in the case of the anterior N2, or a correction, in the case of the ERN. This would predict that correct responses would be slower in trials where the representation of the incorrect response was larger. Burle, Roger, Allain, Vidal and Hasbroucq [53] found such a relationship in a study of partial errors, where the magnitude of the ERN was directly related to the delay from the partial error to correct response execution. However, here we failed to find such a relationship, perhaps because the incorrect response was not fully active in these trials. Thus, activation of the incorrect response might not have had a strong impact on the execution of the correct response in the current study.

One limitation of the current study is that the representation of the correct and incorrect responses and their accompanying stimuli were confounded: the number of arrowheads signaled the number of fingers in a given response. While this facilitated training, it means the effects on dACC activity could be attributed to either the representation of the incorrect response itself, or the flanker stimulus. Forster, Carter, Cohen and Cho [54] suggested that increasing the perceptual information related to the incorrect response would increase response conflict and showed that this manipulation created corresponding increases in correct response RT, error rate, and anterior N2 magnitude. We did not confirm these observations here: increasing the number of arrowheads in the flanker did not consistently increase behavioral signatures of conflict, like correct response RT or error rate. Thus, the manipulation of the flanker stimulus did not appear to induce more response conflict, and therefore is an unlikely explanation for the ERP results observed here. Other work indicates the ERN and anterior N2 respond to erroneous motor tendencies rather than the incongruence of stimuli or the size of the flanker stimulus [20], [52], [55]. Thus, it seems more likely that the effects observed here are related to the motor representation of the incorrect response.

Our findings have implications for monitoring theories of dACC function. In the current study, the similarity of correct and incorrect responses depended on the independent manipulation of response options. Mismatch theories of the ERN suggest that this component indexes detection of an error, and should reflect the degree of mismatch (dissimilarity) between the incorrect and correct responses [56]–[58]. However, no such relationship was found between mismatch and ERN magnitude. In a similar vein, the conflict monitoring model predicts the similarity of the two response alternatives should affect both the ERN and N2 [59]. While the model does not explicitly address cases where there are more than two response options, such as the present task, our results would seem to present a challenge for it. We observed that error rate increased with greater similarity between response alternatives, suggesting that the similarity of target and flanker stimuli affected response conflict. However, response similarity did not appear to affect the ERN or anterior N2. The conflict model would also predict a positive relationship between ERN magnitude and correction frequency [60], while we observed the opposite pattern. Finally, recent accounts suggest that dACC activity is sensitive to time on task [61], [62]. However, we found no relationship between RT and the ERN or anterior N2.

There have been recent efforts to reconcile action selection and decision-making views of dACC function [26], [63], [64]. Our finding that ERP waveforms originating from the dACC reflect rapid within-trial disengagement from an incorrect course of action echoes observations in value-based decision contexts. We show that this disengagement process might operate on a much finer timescale than generally considered in the human decision-making literature. Further work to establish whether the process observed here and that studied in the context of decision-making reflect the same mechanism will be important in developing an appropriately general model of dACC function.
